# Inhibition of Carbonic Anhydrase IX Suppresses Breast Cancer Cell Motility at the Single-Cell Level

**DOI:** 10.3390/ijms222111571

**Published:** 2021-10-26

**Authors:** Agne Janoniene, Linas Mazutis, Daumantas Matulis, Vilma Petrikaite

**Affiliations:** Institute of Biotechnology, Life Sciences Center, Vilnius University, LT-10257 Vilnius, Lithuania; agne.vegyte@bti.vu.lt (A.J.); linas.mazutis@bti.vu.lt (L.M.); daumantas.matulis@bti.vu.lt (D.M.)

**Keywords:** carbonic anhydrase IX, single cell migration, breast cancer, sulfonamide, microfluidic system

## Abstract

Protein Carbonic Anhydrase IX (CA IX), which is expressed in various hypoxic solid tumors in order to maintain proper pH, is also related to cancer cell adhesion, invasion, and metastasis processes. Here, we investigated whether CA IX inhibition by a highly CA IX selective agent benzenesulfonamide VD11-4-2 triggers changes in individual cell motility. We seeded breast cancer cells on an extracellular matrix-coated glass-bottomed dish and in a microfluidic device with a gradient flow of epidermal growth factor (EGF), tracked individual cell movement, calculated their migration speeds, and/or followed movement direction. Our results showed that the inhibitor VD11-4-2 decreased the speed of CA IX positive breast cancer cells by 20–26% while not affecting non-cancerous cell migration. The inhibitor suppressed the cell migration velocity increment and hindered cells from reaching their maximum speed. VD11-4-2 also reduced CA IX, expressing cell movement towards the growth factor as a chemoattractant. Such a single cell-based migration assay enabled the comprehensive investigation of the cell motility and revealed that VD11-4-2 shows the ability to suppress breast cancer cell migration at a lower concentration than previously tested CA IX inhibitors.

## 1. Introduction

Breast cancer is the most common type of cancer occurring in women worldwide (WHO). Although the early stages of breast cancer can be controlled, progressed malignancies or metastases are often associated with poor survival [1]. Therefore, novel therapeutic strategies that would reduce the spreading of cancer cells from a primary site are of great interest and importance [2]. A wide range of agents acting on different targets is being tested in preclinical studies with the purpose of reducing tumor metastasis, but none of them are approved for clinical use [3]. This leads to a search for novel targets that would affect one of the fundamental steps of metastasis.

Various solid tumor cells, including breast cancer, overexpress CA IX protein [4]. This metalloenzyme is trans-membranous with its catalytic site located in the extracellular space. CA IX typically appears under hypoxia and helps cancer cells to survive and proliferate by producing bicarbonate ions, important for maintaining stable intracellular pH [5]. Furthermore, CA IX participates in such metastasis-related steps as cell-cell adhesion, cell interaction with the extracellular matrix, and protrusion formation [6,7,8]. The expression of CA IX is observed to be higher in metastatic areas as compared to the primary tumor site [9]. The knockdown or inhibition of CA IX leads to reduced tumor metastases, as shown during numerous studies in vivo [10,11,12]. In vitro studies with breast cancer cell lines have shown that CA IX inhibitors (ureido sulfonamides and sulfamates) could have the potential to affect cell migration during a scratch assay [11,13,14]. However, these studies monitored cell migration during the scratch assay, which is based on monitoring how monolayered cells occupy the empty cell culturing dish space, while cell metastasis is more commonly a single cell interaction with extracellular matrix (ECM) processes [15,16].

**Figure 1:**
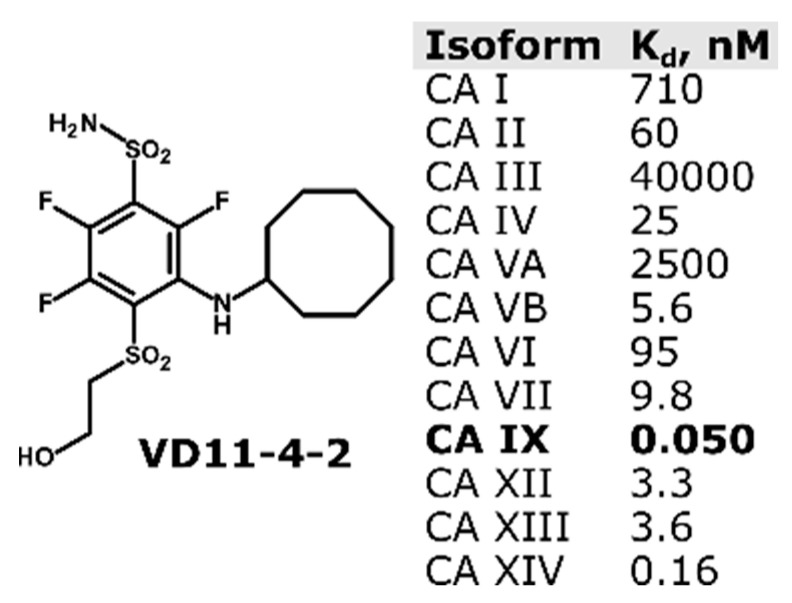
Chemical structure of compound VD11-4-2 and its dissociation constants according to the data provided in [19].

We aimed to investigate the influence of CA IX inhibition on the migration of individual breast cancer cells originating from the metastatic sites and located on collagen-coated surfaces as an ECM-mimicking scaffold. We coated the migration surfaces with collagen and seeded cells mixed with fibronectin in a microfluidic device for closer recapitulation of the physiological conditions of breast cancer sites, which are known to be enriched in ECM components [17,18]. We tracked single-cell movement, translated it into trajectories, and converted it to velocities. Furthermore, we evaluated cell movement towards higher concentrations of EGF used as a chemotaxis stimulant. As a CA IX inhibiting compound, we chose a highly affinitive and selective CA IX inhibitor fluorinated benzenesulfonamide VD11-4-2 (Figure 1) [19]. Previous studies have shown that VD11-4-2 inhibits the function of CA IX expressed in *Xenopus* oocytes and MDA-MB-231 cells in a nanomolar concentration [20]. VD11-4-2 was also low toxic, as shown during experiments with embryonic zebrafish [21]. Thus, VD11-4-2 was previously shown to be highly promising for targeting hypoxic cancer cells through CA IX [22,23].

## 2. Results

### 2.1. CA IX Expression in Cell Lines

**Figure 2:**
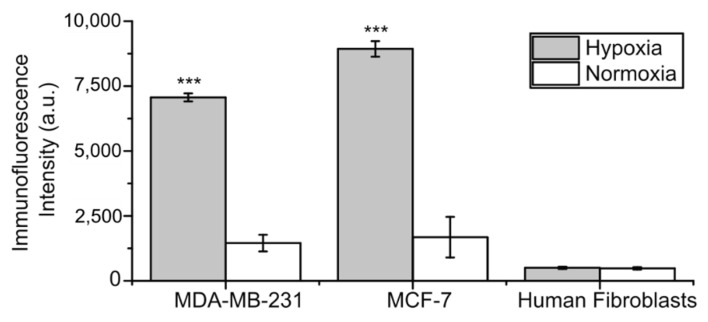
Fluorescence intensities calculated from CA IX immunofluorescence images of MDA-MB-231, MCF-7 cells, and human fibroblasts BJ-5ta under hypoxic as well as normoxic conditions (*** *p* < 0.001).

CA IX expression in breast cells MDA-MB-231 and MCF-7 under hypoxic and normoxic conditions was evaluated by CA IX immunofluorescence. A significantly higher expression of CA IX in both cancer cell lines incubated under hypoxia-mimicking conditions was observed after immunofluorescence staining (Figure S1). This corresponds with observations made by other scientists—that cell treatment with CoCl_2_ as a hypoxia-mimicking compound is suitable for CA IX expression induction in breast cancer cells [24,25]. In contrast, fibroblasts BJ-5ta did not show CA IX expression under any conditions (Figure 2). These results match with findings of previous studies, showing no CA IX expression in fibroblasts derived from human skin [26].

### 2.2. CA IX Inhibitor Suppresses Cancer Cell Velocity

**Figure 3:**
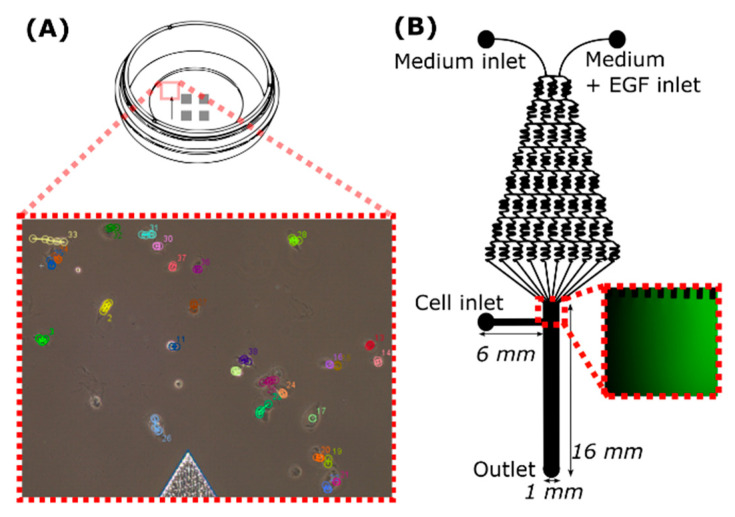
Schematic view of a µ-dish (**A**) with the inset of tracked cells and microfluidic device (**B**) with fluorescence image (inset) of the main channel where the gradient flow of the fluorescein could be seen.

**Figure 4:**
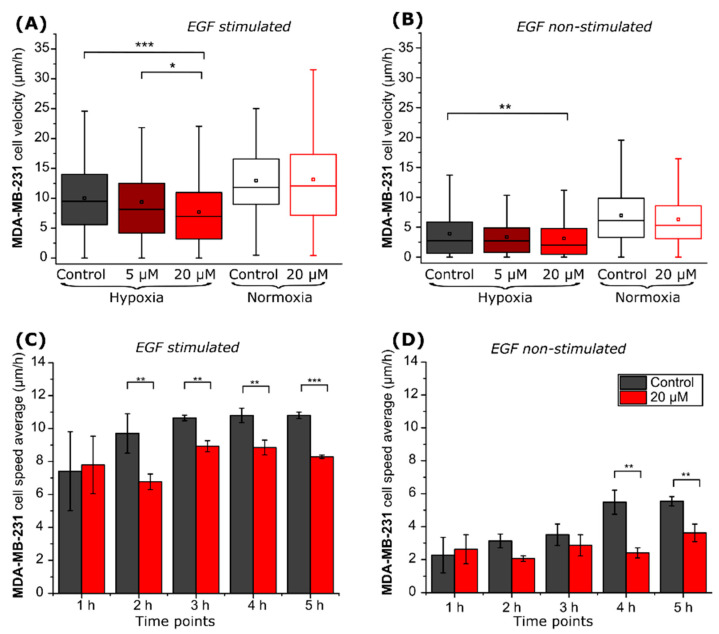
MDA-MB-231 cell migration properties. Control and VD11-4-2 (5, 20 µM) treated MDA-MB-231 cell velocities (**A**,**B**) and hypoxic cell speed changes during the time (**C**,**D**), then cells are stimulated (**A**,**C**) and non-stimulated with EGF (**B**,**D**) (* *p* < 0.05; ** *p* < 0.01; *** *p* < 0.001).

Tracking of individual cells located on a µ-dish (Figure 3A) was used to monitor compound VD11-4-2 (5, 20 µM) influence on cell migration. MDA-MB-231 cell migration speed was lower under hypoxic compared to normoxic conditions. Compound VD11-4-2, at the concentration of 20 µM, reduced hypoxic cell velocity from 10.0 to 7.7 µm/h (*p* < 0.001) in the presence of EGF (Figure 4A), and from 3.9 to 3.1 µm/h (*p* < 0.01) in the absence of EGF (Figure 4B). The compound did not significantly reduce cell velocity at a concentration of 5 µM, as compared to the control. No significant migration changes were observed in normoxic cells incubated with or without the compound. 

Then, we calculated cell velocities at each hour of the experiment. The EGF-treated MDA-MB-231 cells under hypoxic conditions reached a steady-state velocity of 10.6 ± 0.2 µm/h after 3 h of incubation. In contrast, cells lacking EGF stimulation reached a steady state of 5.5 ± 0.7 µm/h after 4 h of incubation. VD11-4-2 prevented EGF-treated and non-treated cells from reaching their maximum velocities, which were 8.9 ± 0.3 and 3.6 ± 0.5 µm/h, respectively (Figure 4C,D).

**Figure 5:**
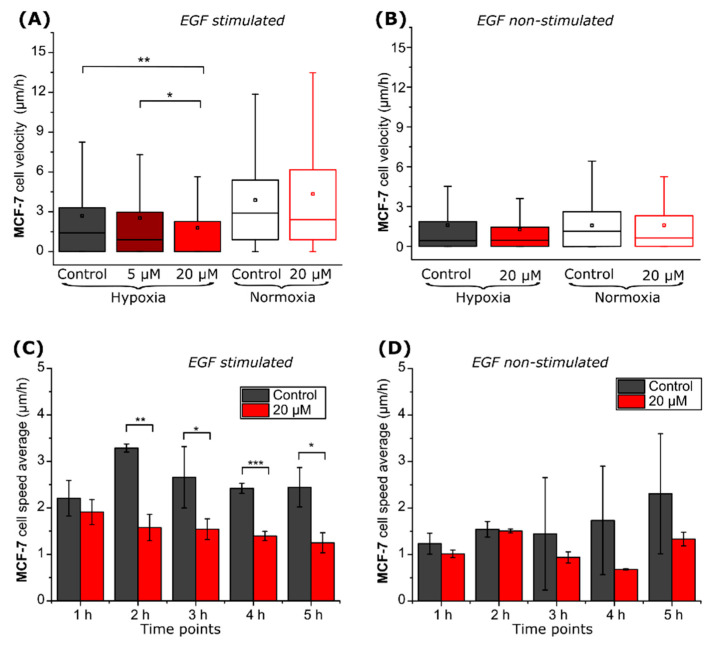
MCF-7 cell migration properties. Control and VD11-4-2 (5, 20 µM)-treated MCF-7 cell velocities (**A**,**B**) and hypoxic cell speed changes during the time (**C**,**D**), then cells were stimulated (**A**,**C**) and non-stimulated with EGF (**B**,**D**) (* *p* < 0.05; ** *p* < 0.01; *** *p* < 0.001).

Afterward, experiments with another breast cancer cell line MCF-7 were conducted. The migration rate of EGF non-stimulated MCF-7 cells was exceptionally low (1.3–2.0 µm/h), and no significant differences were observed between the experimental groups (Figure 5B). To increase velocities, cells were stimulated with 50 ng/mL of EGF (Figure 5A). Statistically significant differences between the control group and the 20 µM VD11-4-2 treated group were observed only in hypoxic cells: the compound reduced the velocity from 2.7 to 2.0 µm/h (*p* < 0.01) (Figure 5A). Compound-hindered EGF-treated and non-treated MCF-7 cells reached their maximal speed during the experimental hours (Figure 5C); however, the latter ones showed no statistical significance (Figure 5D).

VD11-4-2 in a concentration of 20 µM had no significant influence on human fibroblasts cell velocity (Figure S2). Fibroblasts in both control and compound-treated groups were observed to show a maximum in the first experimental hour (6.6 ± 0.08 µm/h for control and 7.03 ± 0.62 µm/h for 20 µM VD11-4-2 treated group).

### 2.3. CA IX Inhibitor Influences Cell Chemotaxis

**Figure 6:**
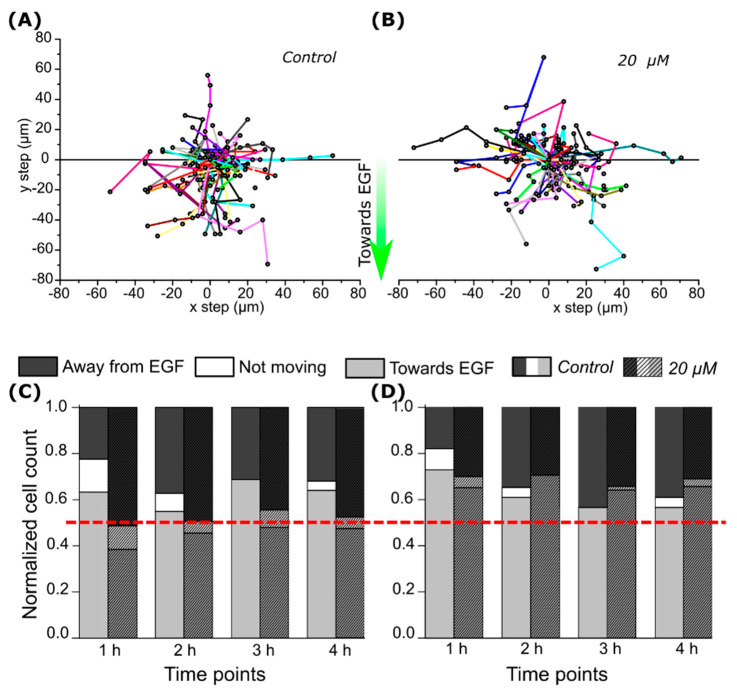
MDA-MB-231 cell chemotaxis. MDA-MB-231 cell migration paths in the control group (**A**) and in a group treated with 20 µM of VD11-4-2 (**B**). Paths towards negative y values show migration towards higher EGF concentrations. Normalized cell count of cells under hypoxia (**C**) and normoxia (**D**) migration towards or away from higher EGF concentrations.

Cell migration direction and speed in the microfluidic device (Figure 3B) were assessed by recording the location of individual cells in the *x* and *y* directions after setting a starting position at the coordinates 0,0 (Figure 6A,B). More than half (>55%, *p* < 0.01) of the control group cells migrated towards greater concentrations of EGF under both hypoxic and normoxic conditions (Figure 6C,D). Inhibitor VD11-4-2 altered CA IX-expressing cells showed attraction towards EGF; no significant differences between cells migrating towards and from higher EGF concentration were observed (Figure 5C). Such reduction of cell migration towards EGF was not observed in CA IX non-expressing normoxic cells treated with VD11-4-2, as the majority (>64%, *p* < 0.001) of cells were moving towards higher EGF concentrations (Figure 6D).

**Figure 7:**
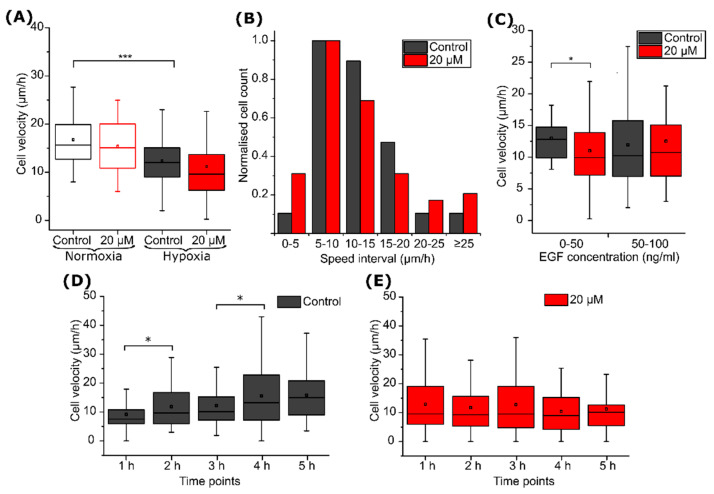
MDA-MB-231 cell migration in the microfluidic device. Velocities of MDA-MB-231 cells (**A**) under normoxia or hypoxia and in the presence or absence of 20 µM VD11-4-2; the amount of the cells migrating in different speed ranges under hypoxia conditions (**B**), starting EGF concentration influence on cell velocities (n = 30, 49, 31, 27), (**C**) and velocity changes during the time of control (**D**) and compound treated (**E**) cells (* *p* < 0.05; *** *p* < 0.001).

Average single-cell speed calculations showed that hypoxia itself reduced cell velocity; in control experiments without compound, the cell velocity was 16.6 ± 1.0 µm/h in normoxia and dropped down to 12.3 ± 2.0 µm/h in hypoxia (*p* < 0.001) (Figure 7A). Cell migration under hypoxia was further investigated by grouping cells according to their migration rate intervals (bins) and normalizing bin values to the maximum. The inhibitor VD11-4-2 caused a three-fold increase in the fraction of the slowest (non-migrating or migrating less than 5 µm/h) cells (Figure 7B). The compound also reduced the number of cells migrating in the speed range of 10 to 20 µm/h. 

We noticed that the VD11-4-2 influence on cell migration was dependent on the initial EGF concentration (Figure 7C). VD11-4-2 reduced cell velocity by almost 2 µm/h (*p* < 0.05) when the starting EGF concentration was from 0 to 50 ng/mL but had no significant effect when the starting EGF concentration was between 50 and 100 ng/mL. No changes in the speed of control group cells under different EGF concentrations were observed.

Finally, exposure to the VD11-4-2 compound also affected cell migration rate profiles. The migration speed of hypoxic cells increased monotonically during the time of the control experiment (Figure 7D); however, no statistically significant increase in cell velocity was observed when 20 µM VD11-4-2 was added (Figure 7E).

## 3. Discussion

CA IX presence in breast cancer patients has been shown to correlate with tumor metastasis and poor prognosis [27,28]. It is known that CA IX localizes in protrusions of migrating tumor cells and participates in reforming its cytoskeleton [29]. Therefore, CA IX inhibitors are being tested for the arrest of cell motility. CA IX inhibiting agents should show high selectivity towards it, as the inhibition of other CA isoforms that are important for non-cancerous cell (such as kidney and red blood cells) functioning would likely cause side effects. In our study, we showed for the first time how highly CA IX affinitive and selective inhibitor VD11-4-2 affects the migration of individual cells located on ECM. The chosen method allows for a comprehensive analysis of cell motility changes during the time of the experiment.

We found that 20 µM of VD11-4-2 diminishes MDA-MB-231 cell velocities. Such a concentration of VD11-4-2 is sufficient to inhibit CA IX functions, as seen from previous studies [20,30]. Moreover, it is not expected to be toxic to cells, as LD_50_ of this compound in zebrafish embryos is determined to be 120 µM [21]. No significant migration velocity changes were observed in normoxic cells treated with VD11-4-2. Previous studies with CA IX inhibitor U-104, which is currently undergoing clinical trials for metastatic pancreatic cancer treatment in combination with gemcitabine, also showed that it could influence CA IX positive (hypoxic) MDA-MB-231 cell migration [10]. However, U-104 reduced the cell migration speed by about 10% at a concentration of 25 µM. Meanwhile, we observed that VD11-4-2 showed a cell migration rate reduction by more than 20% at an even lower concentration of 20 µM. A study by Ward et al. demonstrated that a group of CA IX sulfamate inhibitors influences MDA-MB-231 cell migration in both normoxic and hypoxic conditions [14]. However, influence on migration was measured after a 48 h incubation time, which is longer than cell population doubling time. Single-cell tracking allowed us to observe the hourly changes in cell motility and distinguish the observed effects from those related to cell proliferation. 

VD11-4-2 was further tested by using the MCF-7 breast cancer line, which is also derived from a metastatic site [31]. MDA-MB-231 and MCF-7 differ in their genotypic and phenotypic properties, one of which is receptor expression: while the former is a triple-negative cell line, the latter is estrogen and progesterone positive [32]. However, both show enhanced CA IX expression under hypoxia, and the use of MCF-7 ensured that the effect on migration was not specific to the MDA-MB-231 cell line alone. The formerly mentioned inhibitor U-104 did not influence MCF-7 cell line migration in wound healing assay [13]. Previous studies show that MCF-7 cells have a lower rate of migration and invasion when not stimulated by growth factors [33]. In our study, EGF non-stimulated MCF-7 cell speed was too low to observe statistical differences between the groups. However, we observed that VD11-4-2 (20 µM) significantly reduces EGF stimulated MCF-7 cell migration speed. Human fibroblasts migration that does not express CA IX protein was not affected by VD11-4-2 exposure, therefore indicating that this compound influences only CA IX-positive hypoxic cell migration.

Migration suppression activity of VD11-4-2 was not higher than that reported for cell contractility affecting migrastatic agents [34,35]. However, it should be noted that such cell contractility affecting agents would also alter the migration of healthy cells and, in such a way, could cause damage to the immune system, neuronal process, and tissue rebuilding [36]. Therefore, VD11-4-2 ability to act only on CSAIX-positive cancer cells is an advantageous property.

Next, we investigated VD11-4-2 influence on EGF chemotaxis response of cells located in a microfluidic device with a gradient flow of EGF. Such models are well suited to investigate the molecular mechanisms involved in tumor metastasis [37]. We observed that the majority of control cells migrated towards higher EGF concentrations. This coincides with the observations of Wang et al. that more than half of the cells in a microfluidic channel with 0–100 ng/mL EGF flow migrate towards higher EGF concentrations [38]. The VD11-4-2 compound (20 µM) reduced the fraction of hypoxic CA IX-expressing cells migrating towards the EGF. Cells became non-chemotactic as cell numbers migrating towards and from a higher concentration of EGF became nearly equal. Earlier research showed that the stable expression of CA IX enhances cell chemotaxis towards EGF, while inhibitor U-104 at a concentration of 50 µM was observed to reduce such migration [10].

Single-cell tracking allowed deeper insight into the cell motility properties. Statistically significant differences between control and VD11-4-2-treated hypoxic cells were not observed in experiments with microfluidic devices. However, ranking the cells regarding their speed led us to observe that VD11-4-2 three-fold increased the amount of the slowest cells, migrating at a lower speed than 5 µm/h. We also observed that compound influence on cell velocities depends on the starting cell location in a microfluidic device, where cells occur at different EGF concentrations. The compound also impacts the velocities of the cell, surrounded by 0–50 ng/mL concentrations of EGF. No differences between the speed of control group cells under different EGF concentrations were observed. This coincided with the previous study observations that MDA-MB-231 cell speed is not EGF-dose dependent [39]. VD11-4-2 was also observed to hinder cell speed increment during that time. This coincided with the observations obtained from experiments in a µ-dish.

## 4. Materials and Methods

### 4.1. Cell Culturing

Human breast adenocarcinoma cell lines MDA-MB-231 and MCF-7, human foreskin fibroblasts BJ-5ta were obtained from the American Type Culture Collection (Manassas, VA, USA) and were cultured in Dulbecco’s Modified Eagle Medium (DMEM) (Gibco, Grand Island, NY, USA), supplemented with 10% Fetal Bovine Serum (FBS) (Gibco, Grand Island, NY, USA), 1% Penicillin-Streptomycin (10,000 U/mL) (Gibco, Grand Island, NY, USA) at 37 °C in a humidified atmosphere with 5% CO_2_ and 21% O_2_ until passage number of 20.

### 4.2. Immunofluorescence

MDA-MB-231, MCF-7, and BJ-5ta cells were grown on glass coverslips under normoxia- and hypoxia-mimicking conditions (by adding 240 µM CoCl_2_ (Merck, Kenilworth, NJ, USA) as a hypoxia mimetic agent into the cell growing medium) for 48 h and then fixed with ice-cold methanol for 20 min. Then methanol was aspirated, and cells were incubated with an incubation buffer (PBS-Gibco, Grand Island, NY, USA) containing 2% bovine serum albumin (Sigma-Aldrich, St. Louis, MO, USA) and 2% FBS. Afterward, cells were incubated with 1:200 CA IX-specific monoclonal antibody (mAb) H7 [40] for 1 h, gently washed, and then incubated with goat anti-mouse AlexaFluor 488-conjugated IgG antibody (Life Technologies, Carlsbad, CA, USA) for another 1 h. The nucleus was stained with DAPI (Thermo Fisher, Waltham, MA, USA). Eclipse Ti-U microscope (Nikon, Tokyo, Japan) was used for immunofluorescence imaging. The program ImageJ (National Institutes of Health, Bethesda, MD, USA) was used to evaluate fluorescence intensity (background was subtracted) of FITC labeled antibodies in cells from 3 different images.

### 4.3. Migration in µ-Dish

A glass-bottomed 35 mm µ-dish with an imprinted grid (Ibidi, Gräfelfing, Germany) as a reference was covered with 50 µL of Rattail Collagen diluted to 1 mg/mL with cell culturing medium alkalized to pH = 7.4 with NaHCO_3_ (Carl Roth, Karlsruhe, Germany). Collagen was left to polymerize for 1 h at 37 °C, and then hypoxic, and normoxic cells were seeded (1.6 × 10^4^ per plate) and left to attach overnight. The medium was changed with the fresh one, containing 0, 5, 20 µM VD11-4-2 and 0, 50 ng/mL recombinant human Epidermal Growth Factor (EGF, Invitrogen, Waltham, MA, USA), and images of cells (at least 120 per experimental group for cancerous cells and at least 30 cells for fibroblasts) in 3 different locations from at least 2 different µ-dishes were taken by using EVOS FL Auto Imaging System (Thermo Fisher Scientific, Waltham, MA, USA). Single-cell movements were tracked for 6 h and translated to trajectories (Figure S2A) by the program ImageJ plugin MTrackJ (developed by E. Meijering, Erasmus University Medical Center, Rotterdam, The Netherlands) from images taken every hour for 6 h. Then cell velocities (µm/h) were calculated.

### 4.4. Migration in the Microfluidic Device

Microfluidic device (Figure 3B) was prepared by pouring poly(dimethyl)siloxane (PDMS base) and crosslinker (Dow Corning) mixture (10:1) into the Petri dish with a silicon wafer, degassing it and after its 12 h incubation at 65 °C was peeled off. A 1.2 mm gauge needle was used to punch inlets and outlets into PDMS. Afterward, PDMS was treated with oxygen plasma and bound to the clean glass slide. The microfluidic device was covered with Collagen type I solution (1 mg/mL) through a cell outlet and placed for 1 h at 37 °C. Then, the syringe containing cell medium with 20 µM of VD11-4-2 was connected through the plastic tube to one inlet. The syringe with the medium containing 20 µM of VD11-4-2 and 100 ng/mL of EGF was connected to another inlet. Syringes (1 mL) with the medium were hung above the microfluidic device, and the medium flowed inside the device by gravity. The main channel was carefully filled with MDA-MB-231 cells (≈1 × 10^6^ cells/mL) mixed with fibronectin (10 mg/mL). Cells were left to attach for 1 h, and then images of the main channel were taken every hour for 6 h with Eclipse Ti-U microscope (Nikon, Tokyo, Japan). Medium without inhibitor was used for control experiments. Results obtained from three experiments are presented, where the number of cells was 61 and 78 (compound group) in hypoxia; 64 and 42 (compound group) in normoxia. Cells were tracked by using the same method as in the µ-dish experiment.

### 4.5. Statistical Analysis

Experiments were done in at least three independent measurements. Data are represented as a value mean with standard deviation or as a box plot. A Student’s two-tailed *t*-test was performed using the Microsoft Excel 365 program and was used for statistical evaluation, setting the level of significance at probabilities of * *p* < 0.05, ** *p* < 0.01, and *** *p* < 0.005.

## 5. Conclusions

In conclusion, we have investigated the effect of CA IX inhibitor VD11-4-2 on MDA-MB-231 and MCF-7 cell migration. This fluorinated benzenesulfonamide agent suppressed the migration rate of CA IX-positive breast cancer cells by up to 26% at the concentration of 20 µM and prevented cells from reaching their maximum velocities. The reduced cell migration was recorded after 2 h of exposure to the compound. Furthermore, it reduced the number of MDA-MB-231 cells migrating towards higher EGF concentrations. Overall, CA IX is a promising target for the development of agents for the suppression of hypoxic cancer cell migration. 

## Data Availability

The data presented in this study and underlying raw data are available on reasonable request from the corresponding author.

## Supplementary Materials

The following are available online at https://www.mdpi.com/article/10.3390/ijms222111571/s1.
