# Supplementary material for: Inhibition of Carbonic Anhydrase IX Suppresses Breast Cancer Cell Motility at the Single-Cell Level
Source: Int J Mol Sci. 2021 Oct 26;22(21):11571. doi: 10.3390/ijms222111571 (PMC8584155; doi:10.3390/ijms222111571)
Supplement: Supplementary file 1 [file ijms-22-11571-s001.zip › ijms-1416960-supplementary.pdf]

Supplementary information

Inhibition of CA IX suppresses breast cancer cell motility at single-cell level

AGNE JANONIENE[a], LINAS MAŽUTIS[a], DAUMANTAS MATULIS[a], VILMA PETRIKAITE[a,b]*

[a] Institute of Biotechnology, Life Sciences Center, Vilnius University, Vilnius LT-10257, Lithuania

[b] Institute of Cardiology, Lithuanian University of Health Sciences, Kaunas LT-50162, Lithuania

*Corresponding author e-mail address: VilmaPetrikaite@gmail.com, Tel: +370 686 29383, ORCID: https://orcid.org/0000-0002-4106-5535

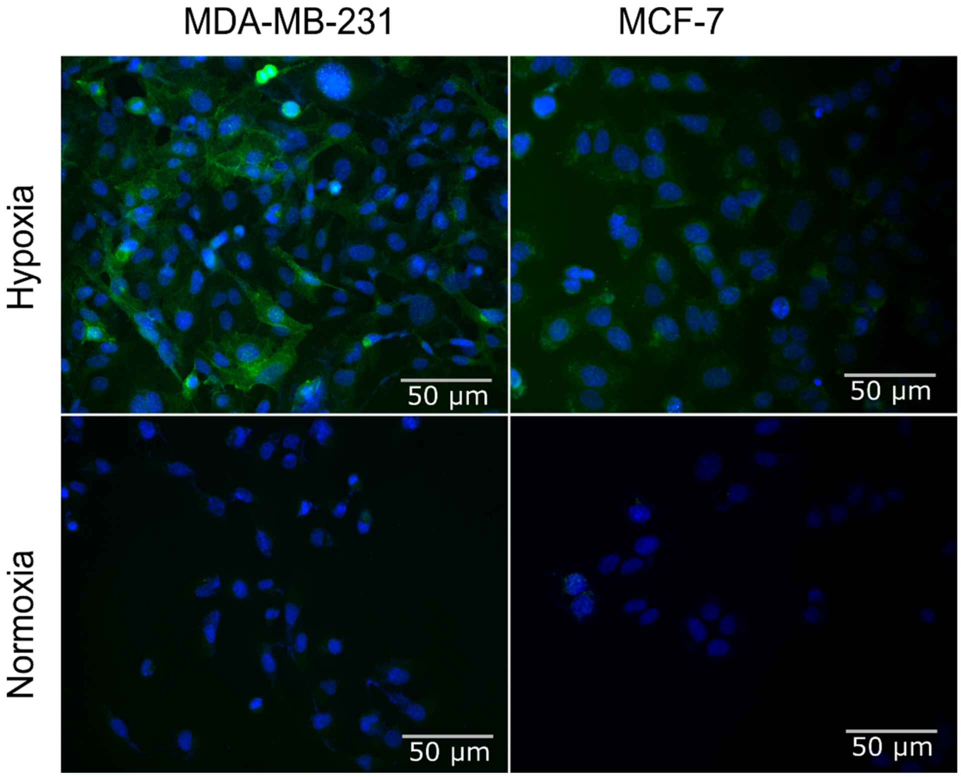

Figure S1. Immunofluorescence images of MDA-MB-231 and MCF-7 cells incubated under hypoxia and normoxia conditions. Nucleus are stained with DAPI (blue) and CA IX is with FITC (green). Scale bar is 100 µm.

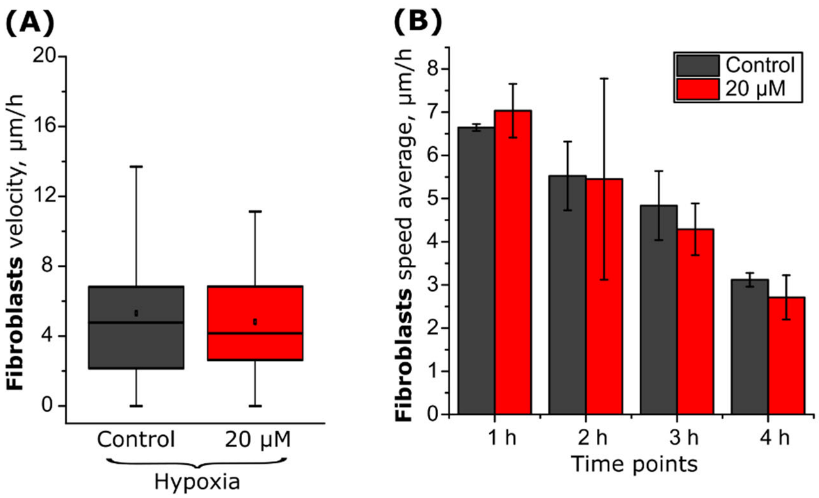

Figure S2. Fibroblasts cell velocities (A) and speed changes during the time (B) in the presence (20 μM) and absence of VD11-4-2 when cells are treated with EGF.
